# COGNITION AFTER A DISASTER: AGE, WORKING MEMORY, AND THE PICTORIAL SUPERIORITY EFFECT

**DOI:** 10.1093/geroni/igae098.2370

**Published:** 2024-12-31

**Authors:** Katie Cherry, Piper Bordes, Katelyn McKneely, Emily Elliott, Matthew Calamia

**Affiliations:** Louisiana State University, Baton Rouge, Louisiana, United States; Louisiana State University, Baton Rouge, Louisiana, United States; Louisiana State University, Baton Rouge, Louisiana, United States; Louisiana State University, Baton Rouge, Louisiana, United States; Louisiana State University, Baton Rouge, Louisiana, United States

## Abstract

We examined free recall of line drawings and matching words in mostly middle-aged and older adults from three parishes (counties) in Baton Rouge, LA that experienced the Great Flood of 2016. All completed multiple individual difference measures, including self-report measures of cognition and emotion (PROMIS, Lai et al., 2014) and a computerized measure of memory for pictures and words. Three groups were compared: (1) non-flooded adults as controls, (2) once-flooded adults with structural damage to homes and property in 2016, and (3) twice-flooded adults who had relocated to Baton Rouge because of catastrophic losses in the 2005 Hurricanes Katrina and Rita and they flooded again in 2016. All were tested during the immediate aftermath of the flood (Wave 1; N = 223, age range: 18-88 years) and most were re-tested 9 +/- 3 months later in a follow-up assessment (Wave 2; N = 202). Results yielded a memorial advantage of pictures over matching words in free recall for all disaster exposure groups at both assessments (p’s < 0.001). Correlation analyses revealed that self-reported deficits in cognition were inversely related to word recall at Wave 1 only. In contrast, self-reported cognitive deficits were positively correlated with flood stressors at Wave 1 and recovery stressors at Wave 2. The present results show that single and multiple disaster exposures do not appear to disrupt performance on traditional, laboratory-based measures of cognition. This research was supported by a grant from the National Science Foundation (Award Number 1708090).

